# Raman Fingerprint of Interlayer Coupling in 2D TMDCs

**DOI:** 10.3390/nano12223949

**Published:** 2022-11-09

**Authors:** Yang Pan, Dietrich R. T. Zahn

**Affiliations:** 1 Semiconductor Physics, Institute of Physics, Chemnitz University of Technology, 09111 Chemnitz, Germany; 2 Center for Materials, Architectures, and Integration of Nanomembranes (MAIN), Chemnitz University of Technology, 09111 Chemnitz, Germany

**Keywords:** 2D materials, TMDCs, 2D heterostructures, Raman spectroscopy, interlayer coupling

## Abstract

Vertical stacking of two-dimensional (2D) homo- and heterostructures are intriguing research objects, as they are essential for fundamental studies and a key towards 2D device applications. It is paramount to understand the interlayer coupling in 2D materials and to find a fast yet precise characteristic signature. In this work, we report on a Raman fingerprint of interlayer coupling in 2D transition metal dichalcogenides (TMDCs). We observed that the out-of-plane $B_{2g}$ vibrational mode is absent when two monolayers form a vertical stack yet remain uncoupled but emerges after strong coupling. Using systematic Raman, photoluminescence (PL), and atomic force microscopy (AFM) studies of WSe${}_{2}$/WSe${}_{2}$ homo-bilayers and MoSe${}_{2}$/WSe${}_{2}$ hetero-bilayers, we conclude that the $B_{2g}$ vibrational mode is a distinct Raman fingerprint of interlayer coupling in 2D TMDCs. Our results propose an easy, fast, precise, and reliable measure to evaluate the interlayer coupling in 2D TMDCs.

## 1. Introduction

The weak van der Waals (vdW) interlayer coupling nature of 2D materials enables the possibility of vertical stacking, which leads to the formation of 2D homo- and heterostructures [1,2]. Novel physics phenomena emerge, and the mechanical, optical, electrical, and magnetic properties can be strongly tuned, when two or more layered materials are coupled to each other, or when the interlayer distance is tuned [3,4,5,6,7,8,9,10,11,12].

Vertical stacking of homo- or hetero-bilayer 2D materials forms moiré superlattices, which induce periodic modulations, potential distribution, phonon renormalization, and lattice reconstruction [13,14,15,16,17,18,19]. The unconventional superconductivity and correlated insulator behavior in magic-angle twisted bilayer graphene opened the door of moiré physics and attracted tremendous research attention to other 2D material systems [20,21]. Moreover, researchers observed interlayer excitons in various combination of TMDC heterostructures such as WSe${}_{2}$/MoSe${}_{2}$, MoS${}_{2}$/WSe${}_{2}$, and WSe${}_{2}$/WS${}_{2}$ [22,23,24], as well as interlayer trions in WSe${}_{2}$/MoSe${}_{2}$/WSe${}_{2}$ heterostructures [25], which builds a promising platform for exploring many-body physics and new optoelectronic phenomena. The coupling of TMDC heterostructures forms pn junctions and can result in type-I, type-II, and type-III band alignment [26], which can significantly widen the application fields and enhance the device performance of transistors, memory devices, light-emitting diodes, photodetectors, and solar cells [27,28,29,30,31,32].

Proper interlayer coupling in 2D homo- and heterostructures is the key to preparing high-quality samples for fundamental studies and achieving high performance in devices. However, the 2D structures are often prepared by dry or wet transfer of mechanically exfoliated or CVD-grown 2D flakes, which always leave impurities consisting of atmospheric gases, water molecules, and hydrocarbons at the interfaces [33]. It is, therefore, paramount to find an easy, precise, and reliable characterization technique to evaluate the interlayer coupling in 2D devices. PL spectroscopy can be used to provide information about the interlayer coupling because of the direct-to-indirect bandgap transition from monolayer to multi-layer TMDCs, the formation of the interlayer excitons, charge transfer, and charge dissociation at the interface all lead to a strong quenching of the PL intensity [3,23,34,35]. However, charge transfer and dissociation can also happen at the interface between 2D materials and the substrates [34]. Moreover, the optical properties of 2D materials are strongly influenced by the substrate [36,37,38], which makes PL spectroscopy not an ideal tool for the characterization of interlayer coupling under certain circumstances, for instance, when 2D materials are supported by a metallic substrate. Raman spectroscopy, on the other hand, is a reliable and non-destructive technique to investigate lattice vibrations and phonon modes of materials. Low-frequency Raman spectroscopy has been intensively used because the interlayer shearing and breathing modes are highly sensitive to interlayer coupling [39,40,41,42,43,44,45]. However, the low-frequency Raman spectroscopy has the drawback of complicated and high-cost optical setup, relatively weak intensity, and disappearing of modes at certain twisting angles [16,41]. Moreover, the low-frequency Raman modes are so sensitive to interlayer coupling that they may also include signals originating from the coupling between 2D materials and hBN, which is commonly used for encapsulation, and thus makes data processing extremely challenging [16,46].

The high-frequency Raman features, on the other hand, are easy to access for almost any conventional Raman spectrometer and do not require extra complicated optical setups. It was reported that the frequency difference between the $E_{2g}$ and $A_{1g}$ vibrational modes for uncoupled artificially stacked bilayer MoS${}_{2}$ is similar to that of monolayer MoS${}_{2}$ rather than intrinsic bilayer MoS${}_{2}$ [45]. However, a distinct high-frequency Raman fingerprint of interlayer coupling in 2D TMDCs has not yet been revealed. In this work, we present a Raman spectroscopy study of interlayer coupling in WSe${}_{2}$/WSe${}_{2}$ homo-bilayer and MoSe${}_{2}$/WSe${}_{2}$ hetero-bilayer as prototypes of homo- and heterostructures. We observe that the out-of-plane $B_{2g}$ vibrational mode is absent in the as-transferred homo- and hetero-bilayers but emerges after annealing. The out-of-plane $B_{2g}$ mode is known to be Raman-active in pristine bilayer WSe${}_{2}$ from the perspective of symmetry [47]. The absence and re-emergence of the $B_{2g}$ signal indicate a significant change in crystal structure and interlayer interaction. Since the samples are prepared on a dielectric substrate (300 nm SiO${}_{2}$/Si) to exclude potential substrate effects, the PL measurements are considered as additional proof for the interlayer coupling. The AFM profiles suggest a significant height change after annealing, which also confirms the coupling. In general, our work demonstrates that the $B_{2g}$ vibrational mode is a Raman fingerprint and an easy, fast, precise, and reliable measure to evaluate the interlayer coupling in 2D TMDCs, which is essential for fundamental studies and device applications.

## 2. Materials and Methods

### 2.1. Sample Preparation

Monolayer WSe${}_{2}$ and MoSe${}_{2}$ were mechanically exfoliated from bulk 2H-phase crystals (WSe${}_{2}$ from HQ Graphene, MoSe${}_{2}$ from 2D Semiconductors) onto polydimethylsiloxane (PDMS) by Nitto tape. The WSe${}_{2}$ homo-bilayer was prepared by a combination of a deterministic all-dry transfer technique and tear-and-stack method [19,48]. A large monolayer WSe${}_{2}$ flake was first exfoliated onto PDMS. The substrate was mounted on a rotation stage. Partial transfer of the monolayer WSe${}_{2}$, rotation of the substrate by 60°, alignment of the flakes, and transfer again leads to an artificial 2H-phase bilayer WSe${}_{2}$. The MoSe${}_{2}$/WSe${}_{2}$ heterostructure was prepared by a deterministic all-dry transfer technique [48]. A detailed description of the sample preparation can be found in the Supplementary Information.

All samples are pre-characterized by PL and Raman spectroscopy on PDMS to identify the layer number. The samples were annealed in a nitrogen atmosphere at 150 °C for 2 h after the initial Raman, PL, and AFM measurements of the as-transferred homo- and hetero-bilayers.

### 2.2. Optical Spectroscopy

Raman and PL spectra were measured in ambient conditions with constant temperature and humidity using a Horiba XploRA${}^{TM}$ Plus Raman Microscope equipped with a 100×, 0.9 NA objective, a spectrometer comprising 600 L/mm (for PL) and 2400 L/mm (for Raman) gratings, and an electron multiplying charge coupled device (EMCCD). A DPSS 532 nm continuous wave (CW) laser source was used to excite the samples with an excitation power of 100 µW measured under the objective. The Raman microscope is equipped with a Märzhäuser motorized xyz stage with a 100 nm step size precision for Raman and PL mapping.

### 2.3. Atomic Force Microscope

We used an AIST-NT SmartSPM${}^{\mathrm{TM}}$ 1000 for AFM measurements. The AFM measurements were performed in ambient conditions with constant temperature and humidity. The NSG10 tip is commercially available with a typical tip radius of ∼6 nm.

## 3. Results and Discussion

### 3.1. WSe${}_{2}$ Homo-Bilayers

**Figure 1:**
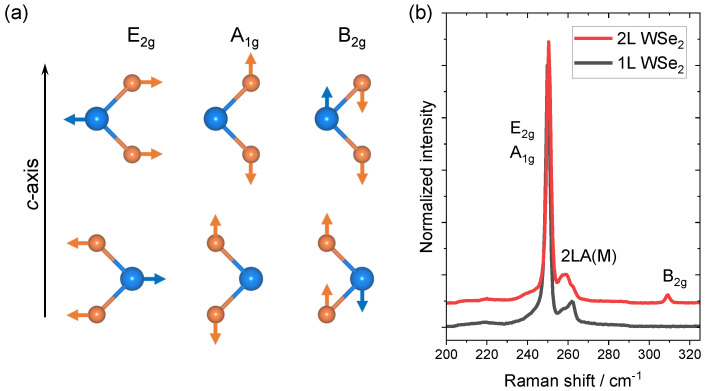
(**a**) $E_{2g}$, $A_{1g}$, and $B_{2g}$ vibrational modes of 2H-phase bilayer WSe${}_{2}$. (**b**) Raman spectra of monolayer and intrinsic bilayer WSe${}_{2}$.

Figure 1a displays the three vibrational modes of WSe${}_{2}$ located in the spectral range of our interest. It is noteworthy that the irreducible representations (irreps) for the same vibrational mode may change in different layer numbers [47], but we refer to these phonon modes by their irreps in the bulk structures to be consistent. The $E_{2g}$ (short for $E_{2g}^{1}$) mode is an in-plane vibrational mode, where the W and Se atoms vibrate against each other. The $A_{1g}$ and $B_{2g}$ modes are out-of-plane vibrational modes. For the $A_{1g}$ mode, the two Se atoms within the same layer vibrate against each other, while the W atom has no relative motion. For the $B_{2g}$ mode, the Se and W atoms within the same layer vibrate against each other with a 180° phase difference to the vibration in the adjacent layers.

From the symmetry point of view, 2H-phase bulk, bilayer, and monolayer WSe${}_{2}$ belong to the $D_{6h}$, $D_{3d}$, and $D_{3h}$ point groups, respectively [49]. The number of symmetry elements is reduced from 24 to 12 when the material is thinned from infinite to few layers. To be clear, though the numbers of symmetry elements are the same in both monolayer and bilayer WSe${}_{2}$, the symmetry elements are actually different. There is one important element present in both bulk and monolayer but missing in bilayer WSe${}_{2}$: a mirror plane in the W atom layer perpendicular to the *c*-axis [50]. The lack of this *c*-axis mirror plane in bilayer WSe${}_{2}$ makes the out-of-plane $B_{2g}$ vibrational mode active [47]. In monolayer WSe${}_{2}$, the existence of a *c*-axis mirror plane and the lack of an adjacent layer makes the $B_{2g}$ mode inactive. The symmetry analysis interprets the Raman spectra in Figure 1b, where the $B_{2g}$ mode at ∼309 cm${}^{-1}$ is absent in monolayer WSe${}_{2}$ while it emerges in bilayer WSe${}_{2}$. The most pronounced peak at ∼250 cm${}^{-1}$ corresponds to the combination of $E_{2g}$ and $A_{1g}$ vibrational modes, almost degenerating at the same frequency [47,50,51,52]. The feature at ∼260 cm${}^{-1}$ is a second-order peak caused by a double-resonance effect involving the longitudinal acoustic phonon at the M point in the Brillouin zone, which is usually assigned as 2LA(M) [52,53]. It is extremely sensitive to the resonance condition due to its double-resonance nature, which explains the different line shapes between monolayer and bilayer WSe${}_{2}$ [53,54].

**Figure 2:**
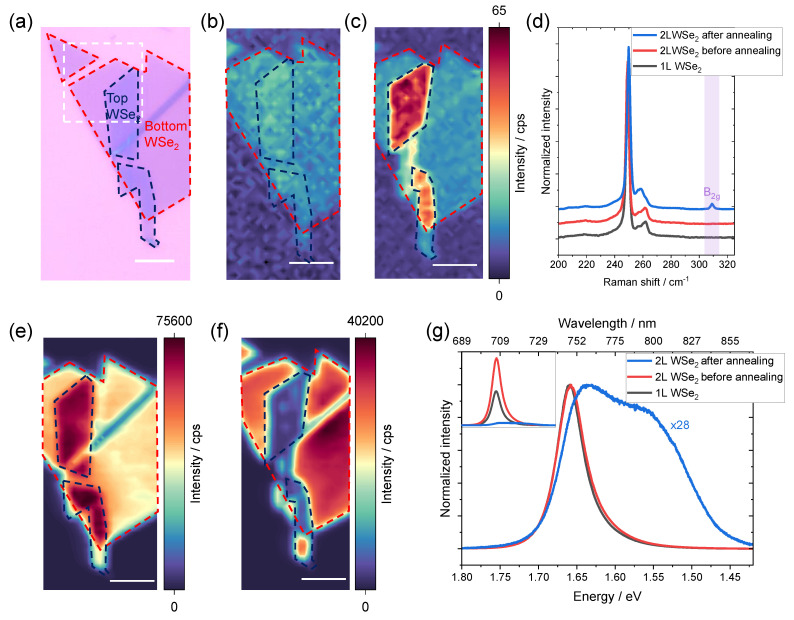
Raman and PL measurements of monolayer and bilayer WSe${}_{2}$. (**a**) Optical microscope image of monolayer and bilayer WSe${}_{2}$. Raman intensity map of the WSe${}_{2}$$B_{2g}$ peak (304–314 cm${}^{-1}$) (**b**) before and (**c**) after annealing. (**b**,**c**) share the same color scale. (**d**) Raman spectra of monolayer and bilayer WSe${}_{2}$ before and after annealing. PL intensity map of WSe${}_{2}$ *A* exciton (1.60–1.75 eV) (**e**) before and (**f**) after annealing. (**g**) PL spectra of monolayer and bilayer WSe${}_{2}$ before and after annealing. Inset: as-measured (not-normalized) PL spectra. The scale bar in figure is 5 µm.

We suggest that the $B_{2g}$ vibrational mode should be a good measure for the interlayer coupling in 2D TMDCs because of the unique property mentioned above. To confirm our hypothesis, we prepared an artificial bilayer WSe${}_{2}$ with a 60° relative twisting angle (shown in Figure 2a) to simulate the situation of the intrinsic bilayer and measured Raman, PL, and AFM before and after the coupling (annealing). It is well known that the interlayer coupling of as-transferred homo- and heterostructures is usually poor [23,33]. Annealing is a practical strategy to improve the interlayer coupling due to the self-cleansing mechanism in vdW heterostructures. The elastic potential energy-induced affinities are higher among the adjacent layers than that between 2D materials and the impurities. The impurities are physically mobile and, therefore, can be driven by the affinity to migrate along the interfaces and finally form isolated bubbles [2,55,56].

Indeed, as shown in the Raman intensity map and spectra in Figure 2b–d, the $B_{2g}$ mode does not exist in monolayer WSe${}_{2}$ and bilayer WSe${}_{2}$ before annealing. However, a clear $B_{2g}$ peak emerges after annealing. The shape of the monolayer flake in the Raman intensity maps in Figure 2b,c originates from the slight change in the background signal. Since a dielectric substrate is used for the sample, the influence of the substrate is negligible in this case. Therefore, PL provides strong additional evidence for the interlayer coupling. As shown in the PL intensity map before annealing in Figure 2e, the PL intensity is extremely high due to the direct bandgap nature of monolayer WSe${}_{2}$, which leads to high radiative recombination efficiency [3,57]. The uncoupled bilayer emits almost twice the intensity as the monolayer part. However, a strong PL quenching is observed on the bilayer part after annealing, as demonstrated in Figure 2f. The strong decrease in PL intensity is caused by the change from direct bandgap in monolayer to indirect bandgap semiconductor in bilayer [3]. An extra emission feature corresponding to the indirect bandgap transition is also observed in Figure 2g. The PL measurements are in excellent agreement with the Raman measurements confirming that the as-transferred bilayer is not coupled, while becoming strongly coupled after annealing. Note that the bilayer area rolled up a bit after annealing along the wrinkle, which causes a slight change in the sample area before and after annealing (as shown in Figure S3 in the Supplementary Information). The comparison of optical microscope images before and after annealing can be found in the Supplementary Materials. All spectra displayed here are taken from the non-rolled-up area.

**Figure 3:**
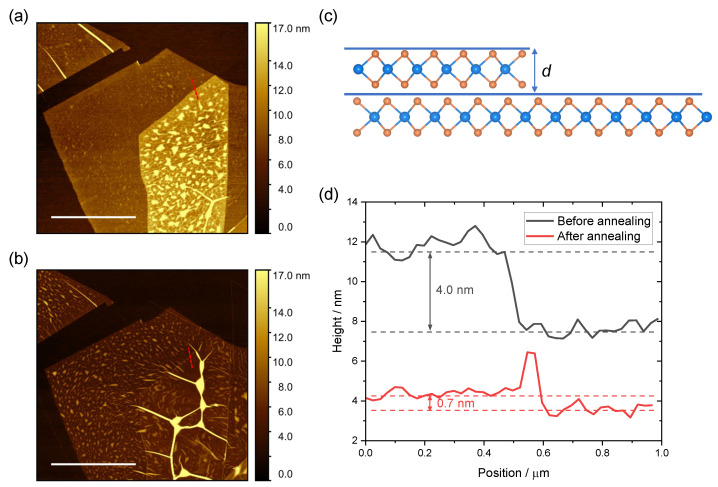
AFM of monolayer and bilayer WSe${}_{2}$ (**a**) before and (**b**) after annealing. (**a**,**b**) share the same color scale. The measured area corresponds to the white squared area in Figure 2a. (**c**) Schematic diagram of the AFM profile. (**d**) Height profile of monolayer and bilayer WSe${}_{2}$ before and after annealing. Scale bar in figure is 4 µm.

In addition to the optical spectroscopy measurements, we also investigated the topography of the samples before and after annealing by means of an atomic force microscope. A clear change in AFM topography is observed. First, the bubbles on the bilayer area in Figure 3a diffuse along the interface and form the big bubbles in Figure 3b. This phenomenon confirms the self-cleaning mechanism in vdW heterostructures. More interestingly, the height profile in Figure 3d suggests that the distance between the top Se atom layer of each individual monolayer decreases from 4.0 nm to 0.7 nm, which agrees with the reported thickness of monolayer WSe${}_{2}$ [58]. The PL and AFM results conclusively confirm that the as-transferred bilayer is not coupled and behaves like two independent monolayers, while becoming tightly coupled after annealing. Combining the Raman, PL, and AFM measurements, it is shown that the $B_{2g}$ mode is a reliable measure for the interlayer coupling in homo-bilayers.

### 3.2. MoSe${}_{2}$/WSe${}_{2}$ Hetero-Bilayers

We demonstrated that the $B_{2g}$ vibrational mode can be considered as a Raman fingerprint to evaluate the interlayer coupling in WSe${}_{2}$/WSe${}_{2}$ homo-bilayers. Furthermore, we investigated the interlayer coupling in TMDC heterostructures using MoSe${}_{2}$/WSe${}_{2}$ hetero-bilayer as a prototype.

**Figure 4:**
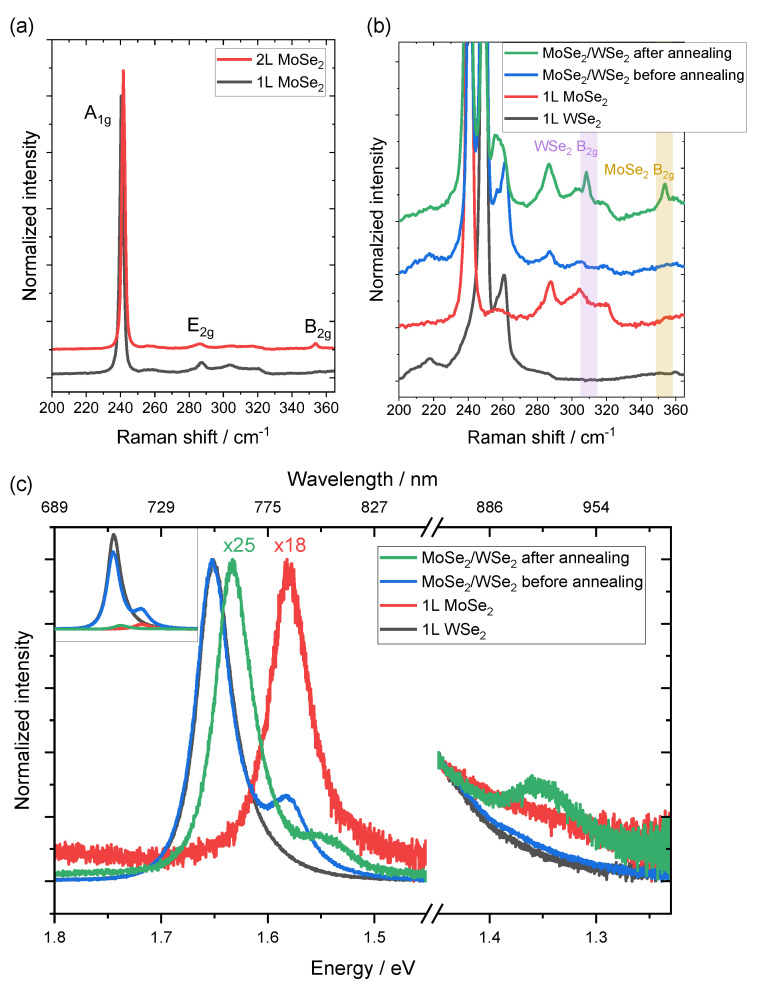
(**a**) Raman spectra of monolayer and bilayer MoSe${}_{2}$. (**b**) Raman and (**c**) PL spectra of monolayer WSe${}_{2}$, MoSe${}_{2}$, and MoSe${}_{2}$/WSe${}_{2}$ hetero-bilayer before and after annealing. The spectra between 1.80 eV and 1.45 eV are normalized from 0 to 1, and the spectra between 1.45 eV and 1.23 eV are normalized from 0 to 0.4 for better visualization. Inset: as-measured (not-normalized) PL spectra.

Figure 4a displays the Raman spectra of monolayer and bilayer MoSe${}_{2}$. MoSe${}_{2}$ has three Raman active modes in the spectral range of our interest, which correspond to the out-of-plane $A_{1g}$ mode, in-plane $E_{2g}$ mode, and out-of-plane $B_{2g}$ mode located at ∼241 cm${}^{-1}$, ∼287 cm${}^{-1}$, and ∼353 cm${}^{-1}$, respectively. Similar to WSe${}_{2}$, the $B_{2g}$ vibrational mode is inactive in bulk and monolayer but becomes active in the bilayer [51].

Figure 4b shows the Raman spectra of monolayer WSe${}_{2}$, MoSe${}_{2}$, and MoSe${}_{2}$/WSe${}_{2}$ heterostructure before and after annealing. The Raman spectra of MoSe${}_{2}$/WSe${}_{2}$ heterostructure before annealing is simply a superposition of the Raman spectra of monolayer MoSe${}_{2}$ and WSe${}_{2}$. Interestingly, both individual $B_{2g}$ modes of WSe${}_{2}$ and MoSe${}_{2}$ emerge after the coupling (annealing). As the key point for the $B_{2g}$ mode to be active is the existence of an adjacent layer and the 180° vibrational phase difference, we propose that the similar structure of TDMCs makes the detection of the $B_{2g}$ mode a fingerprint for bilayer coupling applicable widely for various TMDC combinations. It was reported that the combination of MoSe${}_{2}$ and WSe${}_{2}$ forms interlayer excitons [22], at which point the PL intensity is significantly quenched due to the interlayer charge transfer. Indeed, we observed a ∼25 times PL quenching and an interlayer exciton emission feature at ∼1.35 eV on MoSe${}_{2}$/WSe${}_{2}$ heterostructure after annealing, as shown in Figure 4c, which confirms the proper coupling after annealing. We, therefore, propose that the $B_{2g}$ vibrational mode also serves as a Raman fingerprint for interlayer coupling in TMDC heterostructures.

## 4. Conclusions

In summary, taking WSe${}_{2}$/WSe${}_{2}$ homo-bilayer and MoSe${}_{2}$/WSe${}_{2}$ hetero-bilayer as prototypes, we investigated the interlayer coupling in 2D TDMCs. Using systematic Raman, PL, and AFM, we conclude that the out-of-plane $B_{2g}$ vibrational mode is the Raman fingerprint of interlayer coupling. The $B_{2g}$ mode is absent in an uncoupled vertical stack, but emerges after the coupling. Our work demonstrates an easy, fast, precise, and reliable measure to evaluate interlayer coupling in 2D TMDCs, which is essential for fundamental studies and device applications.

## Data Availability

The data presented in this study are available on request from the corresponding author.

## Supplementary Materials

The following supporting information can be downloaded at: https://www.mdpi.com/article/10.3390/nano12223949/s1, Figure S1: Sample preparation of WSe${}_{2}$/WSe${}_{2}$ homo-bilayer; Figure S2: Sample preparation of MoSe${}_{2}$/WSe${}_{2}$ hetero-bilayer; Figure S3: WSe${}_{2}$/WSe${}_{2}$ homo-bilayer before and after annealing; Figure S4: Raman and PL map of MoSe${}_{2}$/WSe${}_{2}$ hetero-bilayer.
