# Supplementary material for: Raman Fingerprint of Interlayer Coupling in 2D TMDCs
Source: Nanomaterials (Basel). 2022 Nov 9;12(22):3949. doi: 10.3390/nano12223949 (PMC9697269; doi:10.3390/nano12223949)
Supplement: Supplementary file 1 [file nanomaterials-12-03949-s001.zip › nanomaterials-2011669-supplementary.pdf]

Supplementary information: Raman fingerprint of interlayer coupling in 2D TMDCs

Yang Pan[1,2] and Dietrich R. T. Zahn[1,2*]

[1]Semiconductor Physics, Institute of Physics, Chemnitz University of Technology, 09111 Chemnitz, Germany
[2]Center for Materials, Architectures, and Integration of Nanomembranes (MAIN), Chemnitz University of Technology, 09111 Chemnitz, Germany
*Correspondence: zahn@physik.tu-chemnitz.de

1 Sample preparation

1.1 WSe_2/WSe_2 homo-bilayer

WSe_2/WSe_2 homo-bilayer is prepared by a combination of a deterministic all-dry transfer technique and tear-and-stack method [**castellanos2014deterministic**, **Pan2022signature**]. As shown in Figure. **??**, a large monolayer WSe_2 is meachanically exfoliated on PDMS. The monolayer is then partially transferred on a 300 nm SiO_2/Si substrate. The substrate is mounted on a rotation stage and twisted by 60°. The remaining WSe_2 monolayer is aligned and transferred. This will leads to the formation of an artifically-stacked 2H phase bilayer WSe_2.

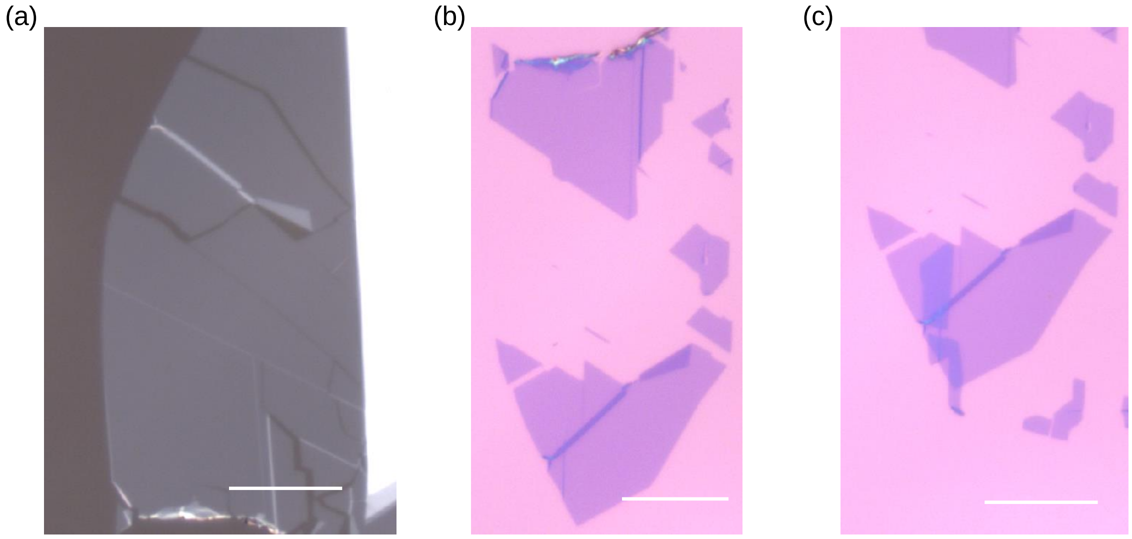

Figure S1: Sample preparation of WSe_2/WSe_2 homo-bilayer. (a) Monolayer WSe_2 on PDMS. (b) Partially transferred monolayer WSe_2 on 300 nm SiO_2/Si. (c) Second transfer with a 60° twisting angle. The scale bar is 15 µm.

1.2 $MoSe_2/WSe_2$ hetero-bilayer

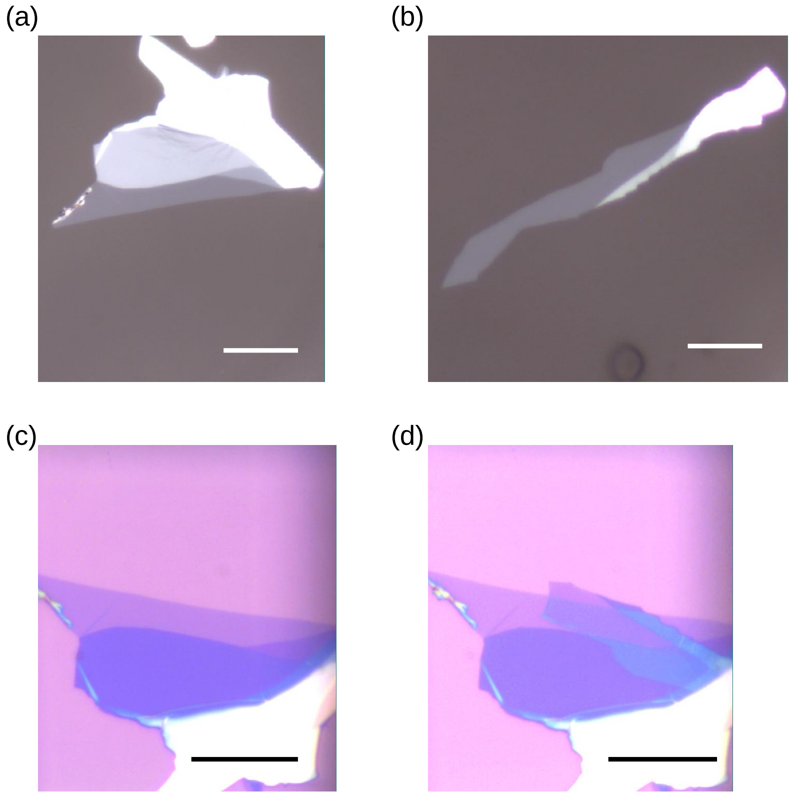

Figure S2: Sample preparation of $MoSe_2/WSe_2$ hetero-bilayer. Monolayer (a) WSe_2 and (b) $MoSe_2$ on PDMS. (c) Monolayer WSe_2 transferred on 300 nm SiO_2/Si. (d) Monolayer $MoSe_2$ transferred on WSe_2. The scale bar is 15 µm.

2 WSe_2/WSe_2 homo-bilayer before and after annealing

The squared area rolled up along the wrinkle after annealing.

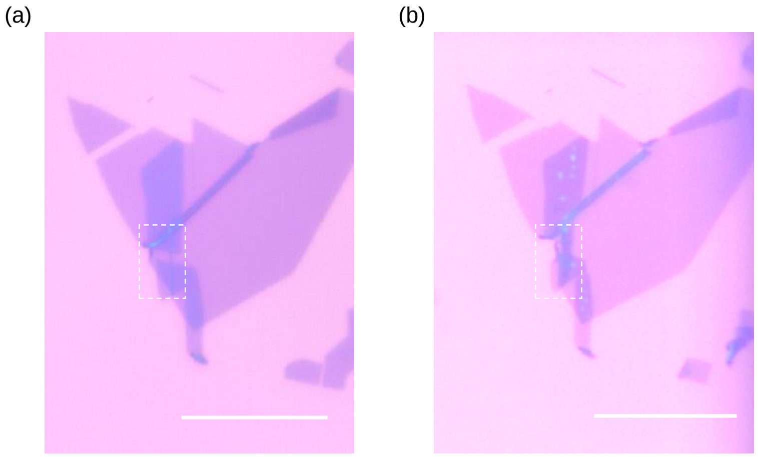

Figure S3: WSe_2/WSe_2 homo-bilayer (a) before and (b) after annealing. The scale bar is 15 µm.

3 Raman and PL map of $MoSe_2/WSe_2$ hetero-bilayer

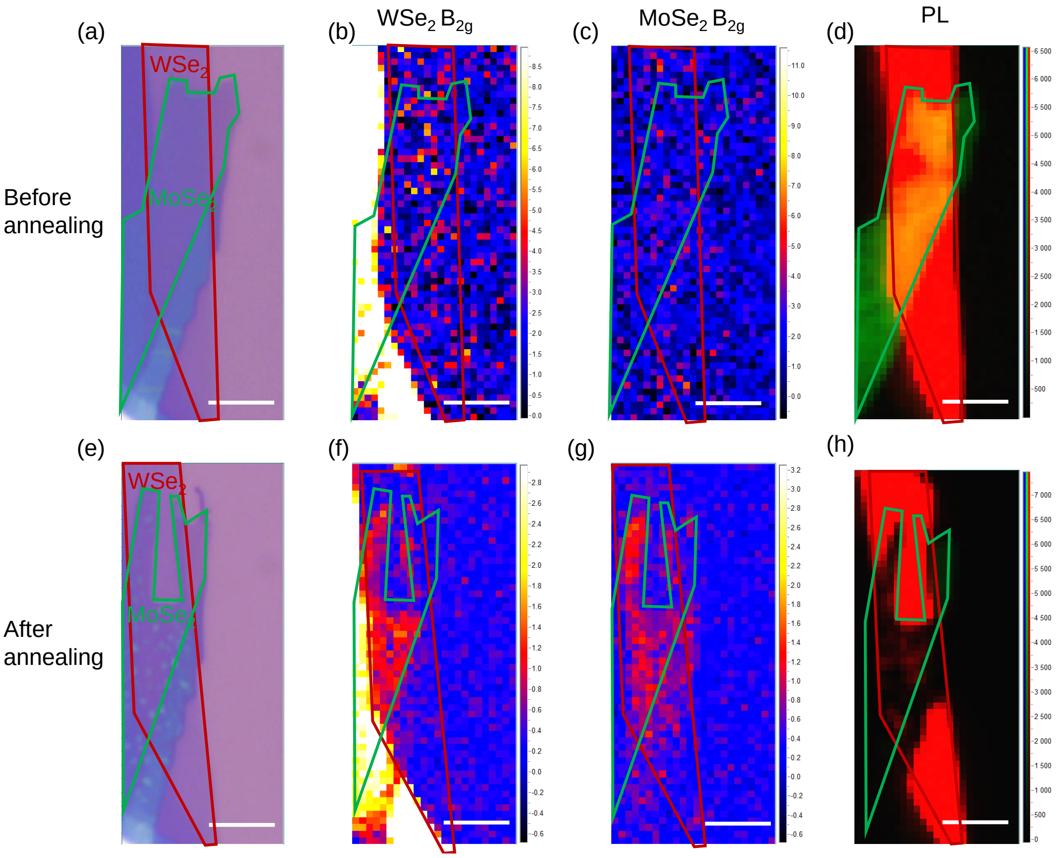

Figure S4: (a) Optical microscope image, (b) WSe_2 B_{2g} Raman intensity map,(c)$MoSe_2$ B_{2g} Raman intensity map, and (d) PL map before annealing. (e) Optical microscope image, (f) WSe_2 B_{2g} Raman intensity map,(g)$MoSe_2$ B_{2g} Raman intensity map, and (h) PL intensity map after annealing. The scale bar is 5 µm.
